# Filamentous Virus-based Assembly: Their Oriented Structures and Thermal Diffusivity

**DOI:** 10.1038/s41598-018-23102-1

**Published:** 2018-04-03

**Authors:** Toshiki Sawada, Yuta Murata, Hironori Marubayashi, Shuichi Nojima, Junko Morikawa, Takeshi Serizawa

**Affiliations:** 1 Department of Chemical Science and Engineering, School of Materials and Chemical Technology, Tokyo Institute of Technology, 2-12-1 Ookayama, Meguro-ku, Tokyo, 152-8550 Japan; 2 Precursory Research for Embryonic Science and Technology (PRESTO), Japan Science and Technology Agency (JST), 4-1-8 Honcho, Kawacughi-shi, Saitama, 332-0012 Japan; 3 Department of Materials Science and Engineering, School of Materials and Chemical Technology, Tokyo Institute of Technology, 2-12-1 Ookayama, Meguro-ku, Tokyo, 152-8550 Japan

## Abstract

Organic polymers are generally regarded as thermal insulators because amorphous arrangement of molecular chains reduces the mean free path of heat-conducting phonons. However, recent studies indicated that single chains of polymers with highly oriented structures could have high thermal conductivity than bulk polymers because stretched polymer chains effectively conduct phonons through polymeric covalent bonds. Here, we demonstrated the possibility of non-covalent virus assembly prepared by simple flow-induced methods toward high thermal conductive polymeric materials. Films with high thermal diffusivity composed of non-covalent bond-based assemblies of liquid crystalline filamentous viruses were prepared using a simple flow-induced orientation method. Structural and thermal characterization demonstrated that highly oriented structures of the viruses in the film were attributed to the high thermal diffusivity. Our results will open attractive opportunities for biomolecular-based thermally conductive soft materials even though the assemblies are based on non-covalent bonds.

## Introduction

Organic polymers with high thermal conductivity have been of great interest for a variety of new, efficient configurations in flexible electronic devices because polymers are lightweight, typically electronic insulators to utilize as films or coatings. However, the thermal conductivity of bulk polymers is very low because of strong phonon scattering caused by various defects, interfaces, and the isotropic molecular orientation in amorphous regions^1,2^. The most common method for improving the thermal conductivity of polymers is often based on the preparation of composite materials, in which additives such as metallic nanoparticles or inorganic nanotubes with high thermal conductivity are embedded in polymeric matrices^3,4^. However, the large amount of such additives (so-called fillers) required to exceed the percolation threshold significantly increases the material cost and may change other unique characteristics, such as electrical and optical properties^5–7^. Therefore, enhancement in the thermal conductivity without using the composite strategy is a challenging and important issue. Several technologies to increase the thermal conductivity of these polymers without fillers include chain orientation improvement^7,8^, self-assembly of monolayers^9^, chain alignment^5,10^, and control of interchain interactions^11^. Typically, highly oriented polymers such as liquid crystalline polymers are promising candidates because the well-oriented liquid crystalline polymer chains effectively conduct phonons through polymeric covalent bonds. In fact, an increase in the degree of orientation in polymers using preciously structural control typically results in a proportional increase in the thermal conductivity against the axis direction of the polymers^5,12^. Although significant efforts have been focused on the development of various technologies to increase the thermal conductivity of polymeric materials, the design of a simple control method to achieve high thermal conductivity via non-covalently assembled structures has rarely gained attention.

M13 bacteriophage (phage), one of the filamentous viruses, is a regular assembly of plural proteins and genetic DNA. Recently, it has been reported that the phages can be utilized as a material component in various fields due to their capabilities of molecular recognition^13–19^, catalysis^20,21^, and nanoparticle nucleation^22,23^. These functions are easily integrated into the phages by genetic^24^ or chemical^25^ modification of the coat proteins on surfaces. Furthermore, the frameworks of phages are competent liquid crystal mesogens that are capable of forming various phases due to their high aspect ratio (4.5 nm width and 900 nm length), dipole properties, and charge densities^26,27^. The liquid crystalline structures are controlled using various methods, and, in particular, a method using substrates (i.e., solid/liquid interfaces) has recently been utilized to create liquid crystalline virus materials, such as sensors, electronics, and devices^28–39^. Based on their well-ordered assembled structures, these virus-based materials, which have diverse properties, can facilitate advances in the effective functionalization for future utilization in new fields.

**Figure 1:**
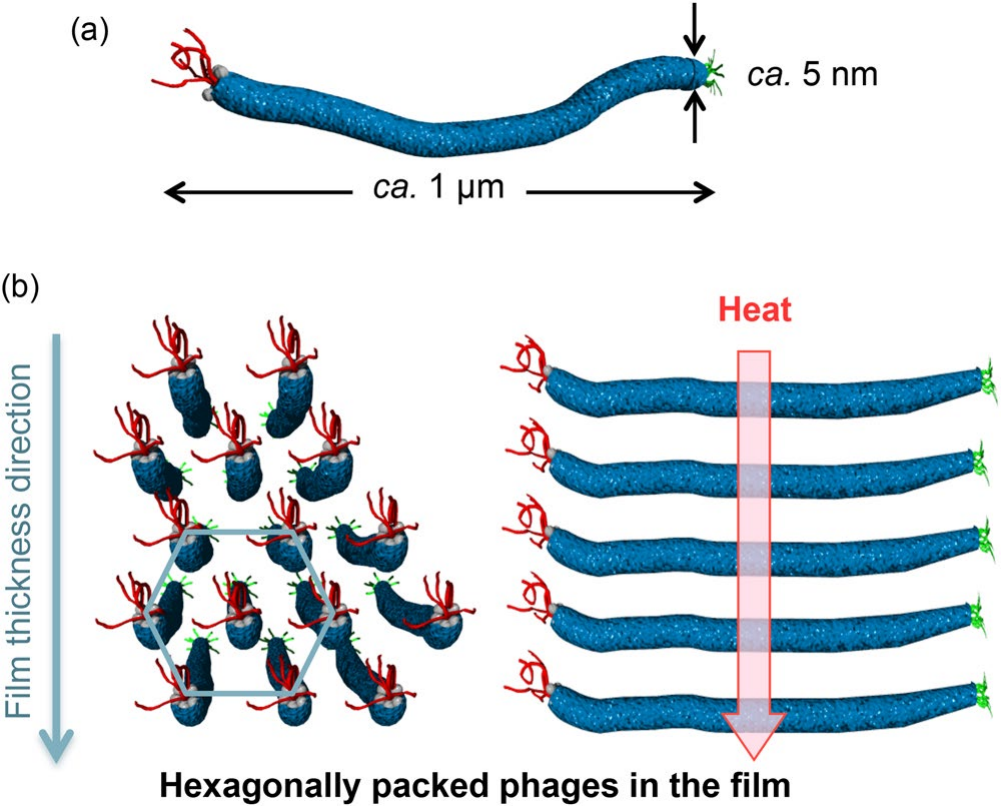
Schematic representations. (**a**) Phage and (**b**) hexagonally assembled structures of the phages in the film.

Although polymer orientation typically results in a proportional increase in the thermal conductivity against in the chain direction of the polymers^5,10^, extension of the persistence length of covalent bonds in the orientation has been the primary focus^7,40^. Although a phage comprises a supramolecular assembly of well-packed coat proteins, the phage acts as a potential thermal conductive material component due to its filamentous structure and liquid crystallinity. Herein, we demonstrated the high thermal conductivity of liquid crystalline films composed of non-covalently assembled phages (Fig. 1). Thermal diffusivity measurements using temperature wave analysis revealed that the films with highly oriented structures exhibit high thermal diffusivity values even though the measurement direction was perpendicular to the filamentous phages. Structural characterization by small-angle X-ray scattering (SAXS) indicated that films with well-packed and highly oriented structures are essential for the high thermal diffusivity even though the assemblies were based on non-covalent bonds. These results provide insight into the relationship between the oriented structures and the thermal conductivity in polymeric materials and provide attractive opportunities in the science and engineering of next-generation thermal conductive soft materials composed of non-covalently assembled structures.

## Results

**Figure 2:**
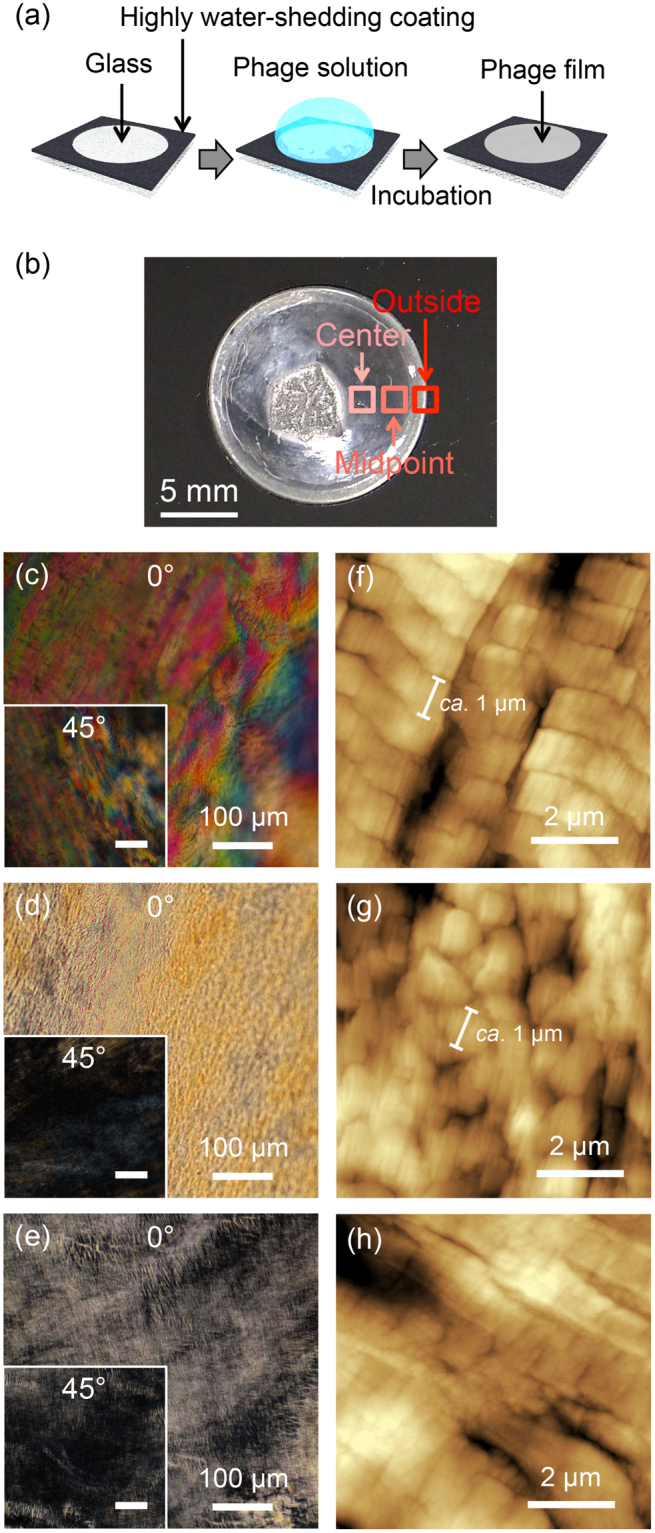
Preparation and microscopic observation of the films. POM images of the (**a**) Schematic illustration of preparation of the phage film. (**b**) Optical photograph of the phage films. The position was indicated in the photograph. (**c–h**) Microscopic observation of the films prepared by flow-induced orientation methods. POM images of the (**c**) outside, (**d**) midpoint, and (**e**) center of the films. Insets represent images after a 45° rotation. AFM images of (**f**) outside, (**g**) midpoint, and (**h**) center of the films. Scale bars are shown in the images.

We demonstrate a straightforward technique for the preparation of phage-films with highly oriented liquid crystalline structures using flow-induced methods and glass plates circularly patterned with a highly water-shedding coating based on fluororesin (leftmost in Fig. 2a). A phage solution (500 µL) with a concentration of 10 mg/mL was mounted on the glass plate (φ: 15 mm) followed by incubation for 24 h to prepare the phage films (Fig. 2a). The thickness of the outside of the film (approximately 1 mm from edge) was visually thicker than other positions on the film. In fact, the film thickness measured by differential transformer methods revealed that the values of the outside, midpoint (between outside and center), and center (Fig. 2b) were 194.0 ± 39.8, 41.9 ± 7.3, and 41.3 ± 7.4 µm, respectively. Because a drop of solution on a solid surface leaves a dense ring-like deposit along the perimeter (coffee-ring effect)^41^, the thicker value on the film outside may be caused by the capillary flow of solutes. Therefore, the outside of the phage films may form via the same manner in solution, whereby it effectively condenses compared to other positions.

Polarized optical microscopy (POM) was performed to characterize the ordered structures of the phage assemblies in the film. On the outside of the film, layered birefringence was clearly observed (Fig. 2c). In general, birefringence is observed in oriented structures of solutes or crystalline molecules^42^. Therefore, phages on the outside of the film have highly oriented structures. When the sample was rotated by 45°, the bright domains became darker, and the dark domains brightened (Fig. 2c, inset), demonstrating the formation of layered structures as smectic liquid crystals. In addition, birefringence without layered structures was observed at the other positions and was darkened by a 45° rotation, suggesting that the structures consist of nematic liquid crystals (Fig. 2d,e). The brightness of the birefringence at the midpoint position of the film was higher than that at the center, indicating a higher ordered structure at the midpoint than at the film center. When the phage films were prepared by the casting method using conventional petri dishes and the same phage concentration, birefringence was rarely observed, indicating the presence of non-oriented structures (Supplementary Fig. S1). Therefore, the phage films prepared using the simple flow-induced method on a patterned glass slide possessed different oriented structures that depended on their position.

The assembled structures of the phages were characterized by atomic force microscopy (AFM). In a height image of the outside of the film, the assembled structures (i.e., smectic liquid crystals) were composed of bending domains with a width of approximately 1 µm (Fig. 2f). The width of the domain was approximately 1 µm, which corresponded to the length of the phage and indicated that the phages were oriented in the same direction in the domains. The phase images of the same region supported the phage oriented and layered structures on the outside of the film (Supplementary Fig. S2a). At the midpoint of the film, domains with a width of 0.5~1 µm and a length of approximately 1 µm in the form of nematic liquid crystals were observed (Fig. 2g and Supplementary Fig. S2b). Furthermore, phage-oriented structures without the formation of domains were observed at the film center (Fig. 2h and Supplementary Fig. S2c), supporting the lower orientation degree of the phages. These microscopic observations suggest that the phages in the film were oriented in a plane direction and that the orientation regularity was higher for the outside of the film.

**Figure 3:**
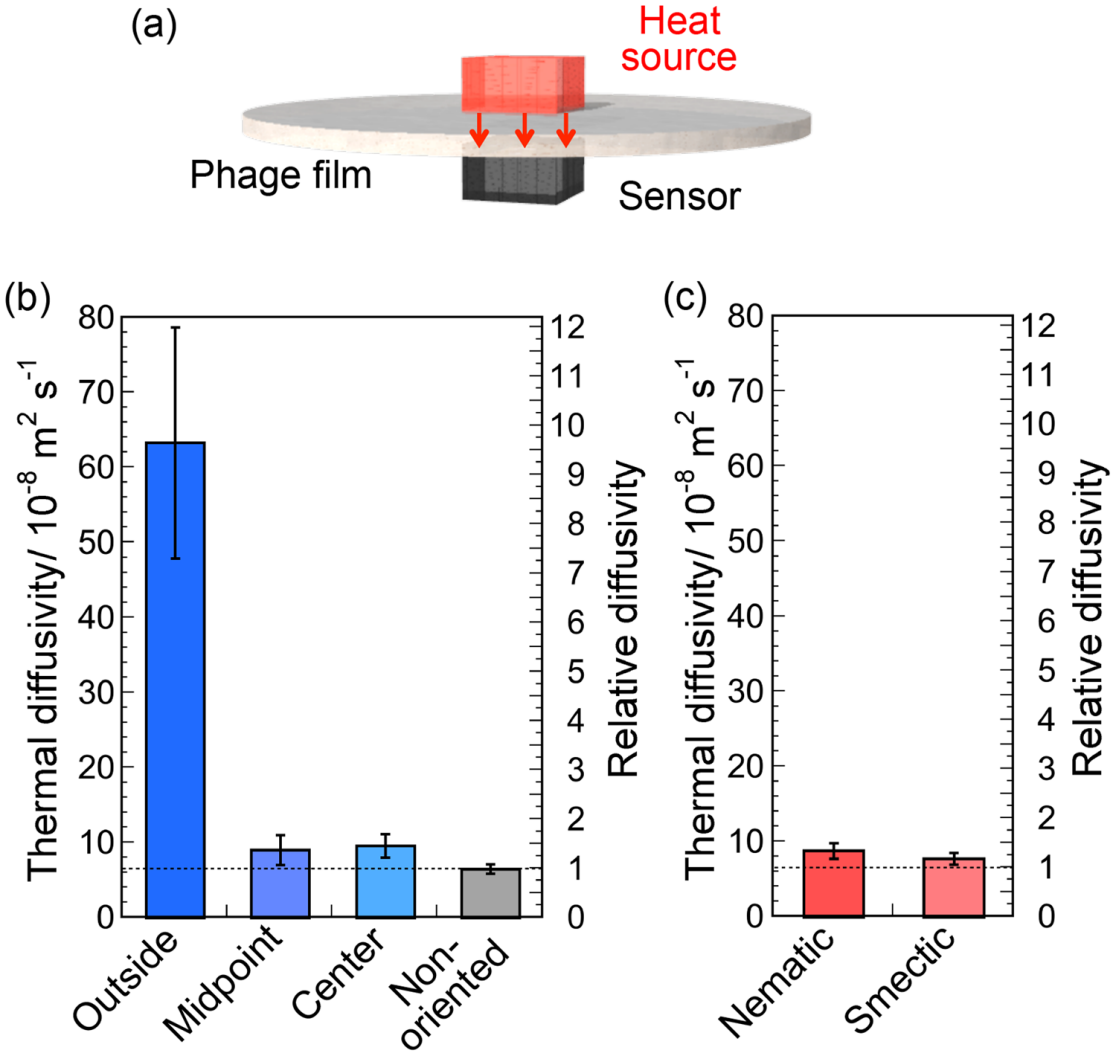
Thermal diffusivity of the phage films. (**a**) Schematic illustration of thermal diffusivity measurements of the phage film. (**b**) Thermal diffusivity of three different positions of the phage films and non-oriented phage films. (**c**) Thermal diffusivity of previously reported liquid crystalline-oriented phage films. Thermal diffusivity values were converted to diffusivities relative to that of non-oriented phage films.

Thermal diffusivity values of the phage films at the outside, midpoint, and center of the film in a perpendicular direction were measured by temperature wave analysis (Fig. 3a and Supplementary Fig. S3). Thermal diffusivity values at different points of the films are summarized in Fig. 3b. The thermal diffusivity value on the outside (6.3 × 10^−7^ m^2^ s^−1^) was approximately 10 times greater than that of the non-oriented film (6.6 × 10^−8^ m^2^ s^−1^) and approximately 7 times greater than those at other points (values at the midpoint and center were 9.1 and 9.6 × 10^−8^ m^2^ s^−1^, respectively), demonstrating the extremely high thermal diffusivity on the outside of the film. The value on the outside is comparable to the value of inorganic glass^43^ and a strand of human hair measured against the axis direction^10^.

**Figure 4:**
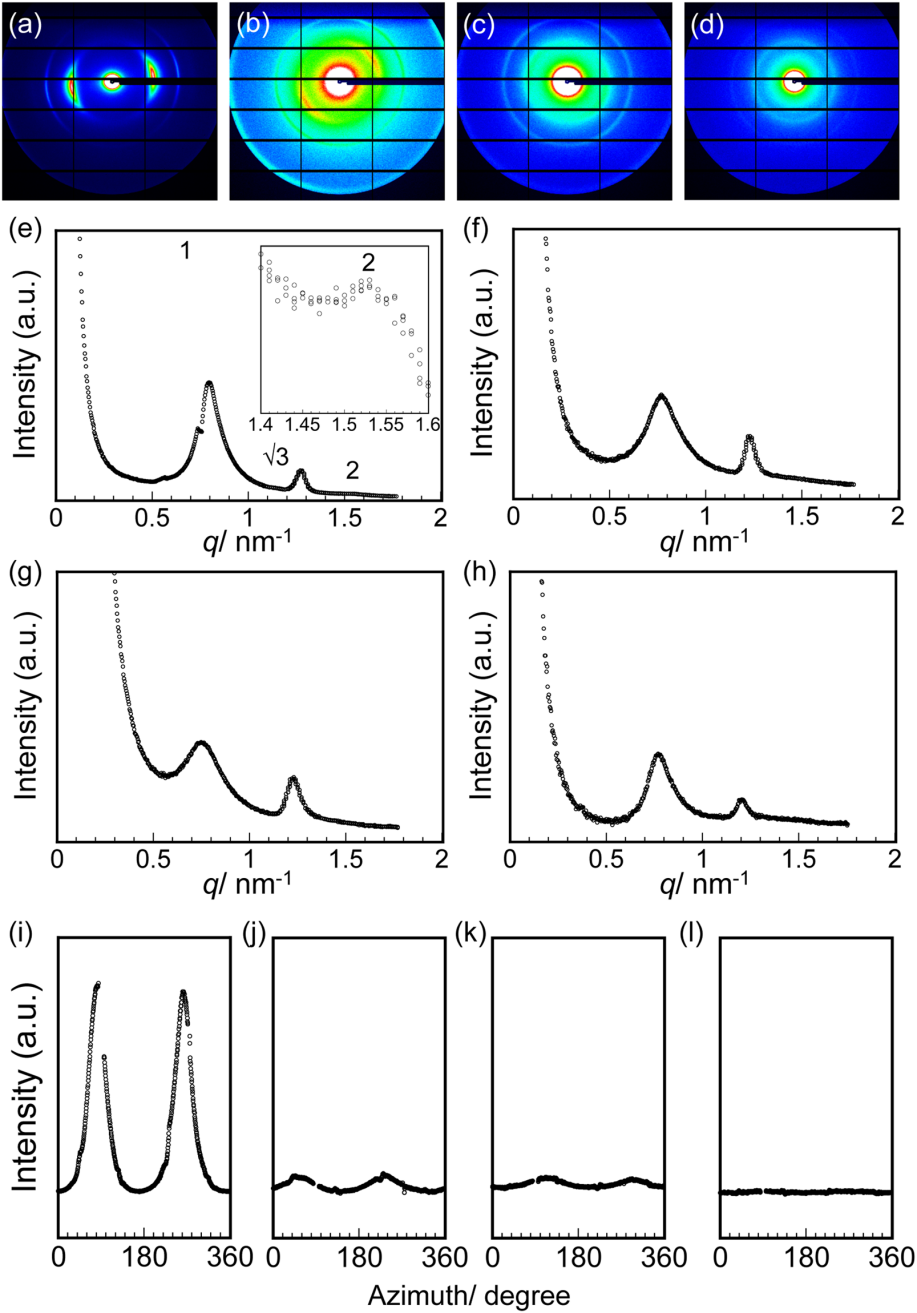
SAXS analyses of phage films. Two-dimensional patterns for the (**a**) outside, (**b**) midpoint, and (**c**) center of the phage films and (**d**) the non-oriented phage films. The scattering curves for the (**e**) outside, (**f**) midpoint, and (**g**) center of the phage films and (**h**) the non-oriented phage films, where intensity was normalized by a film thickness. Azimuth scan of the primary peaks for the (**i**) outside, (**j**) midpoint, and (**k**) center of the phage films and (**l**) the non-oriented phage films. Azimuth is zero in the upper direction from the center and increases in the clockwise direction in 2D SAXS images.

Determining the relationship between the thermal diffusivity and the assembled structures of phages is important for understanding the increase in the thermal diffusivity. As observed by the POM and AFM examinations, the oriented structures of the phages were dependent on the positions, and the outer side of the film exhibited a high orientation degree. To further characterize the oriented structures of the phages, SAXS experiments were performed on the outside, midpoint, and center of the film as well as the non-oriented film (Fig. 4a–d). In the resulting scattering profile for the outside of the film (Fig. 4e), intense peaks at 7.90, 4.92, and 4.01 nm were observed with a reciprocal *d*-spacing ratio of 1:√3:2, which indicates hexagonally packed structures of the phages (Table 1). The scattering profiles for the midpoint and center of the film as well as the non-oriented film composed of randomly assembled phages were characteristic of hexagonally packed structures (Fig. 4f–h). The primary peak intensities and positions (7.9–8.3 nm) were comparable irrespective of the positions, so that packing of the phages on a molecular level was essentially the same.

**Table 1:** Summary of the SAXS measurements of the phage films.

Film	*d*_1_/nm	*d*_2_/nm	*d*_3_/nm	*q*_1_/q*	*q*_2_/q*	*q*_3_/q*	FWHM	Degree of orientation
Outside	7.90	4.92	4.01	1.00	1.61	1.97	39.6	0.78 ± 0.05
Mid point	8.14	5.10	—	1.00	1.60	—	145.4	0.19 ± 0.01
Center	8.25	5.09	—	1.00	1.62	—	178.3	0.01 ± 0.006
Non-oriented	8.10	5.18	—	1.00	1.57	—	—	—

To quantitatively investigate the oriented structures of the phages, an azimuth scan of the primary peaks at approximately 8 nm was performed (Fig. 4i–l). Full width at half maximum (FWHM) was determined by fitting with the pseudo-Voigt function to calculate the degree of orientation, which indicates the orientation of the phages in entire film, as in Equation 1.

1 $$Degree\,of\,orientation=\frac{180-FWHM}{180}$$

In this calculation, the degree of orientation ranges from 0 to 1, and a higher value represents a higher degree of orientation. The resulting degree of orientation of the outside of the film (0.78±0.05) was extremely high compared with those of the other positions (0.19 ± 0.01 and 0.01 ± 0.006 for midpoint and center, respectively), suggesting that the assembled phages on the outside of the film are highly oriented. The degree of orientation for the non-oriented film with the lowest thermal diffusivity could not be determined because no peaks for the azimuth scan were detected.

To gain additional insight into the relationship, previously reported phage films with nematic and smectic liquid crystalline-oriented structures, which were prepared in microtubes using flow-induced methods^28^, were used for subsequent experiments (Supplementary Fig. S4). The degree of orientation of the previous films with nematic and smectic liquid crystalline orientation determined by SAXS experiments was 0.54 ± 0.03 and 0.61 ± 0.02, respectively, and the values were in the range between the film outside and midpoint (i.e., moderate orientation). The thermal diffusivity values of these films were approximately 7 times smaller than that for the outside of the film (Fig. 3b), indicating the importance of the extremely high degree of orientation for high thermal diffusivity.

## Discussion

Because the POM and AFM observations demonstrated that the phages in the film were oriented in the plane direction, it was suggested that the substantial increase in the thermal diffusivity on the outside was not caused by the orientation of the phage being perpendicular to the film plane as well known chain orientation effects of conventional polymers^5,10,11^. Therefore, the differences in the increase in the thermal diffusivity values may be derived from the difference in the orientation of the phage molecules or assemblies even though the thermally measured directions were not in the axis direction of the assembled phages. On the other hand, SAXS measurements demonstrated that the phages in the films would partially form well-packed hexagonal structures through solvent evaporation processes that are independent of the positions even though the macroscopic assembled structures were different. Therefore, the thermal diffusivity was not caused by packing of the phages on a molecular level but predominantly associated with macroscopic phage orientation (that is, orientation of the phage assemblies) in the film to decrease phonon scattering at structural defects. The measured area determined by SAXS (~0.5 mm^2^) was much larger than the domain size of the liquid crystalline-assembled phages observed using AFM (~several µm^2^); therefore, a high value of the degree of orientation indicated that the phage-assembled domains, of which scale was several hundred µm, was highly oriented. In addition, the phages formed well-packed structures with rare defects (i.e., the proteins constituting the phages were very close in distance and packed without gaps). A comparison of the thermal diffusivity and the value of orientation degrees indicated that no linear correlation existed between them, and only films with a high degree of orientation (i.e., 0.81) exhibited high thermal diffusivity. These results suggested that the highly oriented structures of the assembled phages with a scale of several hundred µm resulted in a high thermal diffusivity, which may be due to highly efficient phonon transport. Importantly, the thermal diffusivity values of the previously reported films were comparable to those of the film midpoint and center even though the degree of orientation was very different. Therefore, the increase in the thermal diffusivity was not substantially influenced by the moderate orientation of the domains of the assembled phages. Based on all the structural and thermophysical characterizations, the thermal diffusivity of the liquid crystalline phage film measured against the perpendicular direction substantially increased due to the extremely oriented assembly of the phages, which may be due to a decrease in phonon scattering at structural defects in the phage-assembled structures. These results suggest that well-packed assemblies of phages with few defects play an important role in efficient phonon transport to achieve high thermally conductive soft materials even though the assemblies are based on non-covalent bonds.

## Conclusions

We prepared filamentous phage-based liquid crystalline films and investigated the relationship between their thermal diffusivity and assembled structures. The microscopic observations of the phage films that were prepared by flow-induced orientation methods on glass plates patterned with a highly water-shedding coating revealed that the orientation of the assembled structures differed depending on the positions. The thermal diffusivity value of the outside of the film was approximately 7 times greater than those at the other points on the film and 10 times greater than that of the non-oriented cast film, and an extremely high thermal diffusivity was observed on the outside even though the assemblies were based on thermally-insulating non-covalent bonds. The SAXS analysis to quantitatively characterize the orientation of phages using various phage films indicated that assemblies with highly oriented structures resulted in a high thermal diffusivity in the perpendicular direction of the filamentous phages, which may be due to a small number of structural defects. The great applicability of non-covalent bond-based assemblies will unlock novel and interesting opportunities for exploitation of next-generation thermally conductive soft materials composed of regularly assembled organic polymers such as proteins.

## Methods

### Preparation of phages

The phages were expressed using the Ph.D. Peptide Display Cloning System (New England Biolabs, Inc.). Phagemid DNA was heat-shocked into competent *Escherichia coli* (*E. coli*) ER2738 cells. The expressed phages were amplified using the cells and purified by precipitation and re-dispersion procedures using 5% (w/v) PEG and 2.5 M NaCl.

### Preparation of phage films

Phage solutions (15 mg/mL, 500 µL) were mounted on glass plates circularly patterned with a highly water-shedding coating based on fluororesin (glass diameter: 15 mm, Matsunami Glass Ind., Ltd.) followed by incubation for 24 h at 25 °C in a dry atmosphere. When the phage films were prepared according to previously reported methods^28^, the phage solutions (5 or 10 mg/mL, 400 µL) were added to a conventional poly(propylene)-based 1.7 mL microtube and incubated for 7 d, resulting in phage films formed on the wall of the microtubes.

### POM observation

Phage films were mounted onto the stage of a polarized optical microscope (Eclipse LV100ND, Nikon). Then, the samples were observed at ambient temperature. Images with a sample rotation of 45° were recorded to identify the liquid crystalline-oriented structures.

### AFM observation

Phage films were mounted onto the stage of an atomic force microscope. Then, the samples were observed. The AFM images were obtained using an SPM-9600 (Shimadzu) in tapping mode with standard silicon cantilevers. All images were scanned at a scan rate of 1 Hz with a maximum number of pixels (512 × 512).

### Thermal diffusivity measurements

The thermal diffusivity of the films in the thickness direction was measured using an ai-Phase mobile 1 u (ai-Phase Co. Ltd.) based on temperature wave analysis methods^44^. The thermal diffusivity (α) was calculated from the relationship of the square root of the angular frequency (√ω) and the phase delay (∆θ) of the temperature wave as shown in the following equation,

2 $${\rm{\Delta }}\theta =\,-\sqrt{\frac{\omega }{2\alpha }}d-\frac{\pi }{4}$$

where *d* is the thickness of the film.

### SAXS measurements

SAXS measurements of the phage films were performed at BL-10C (λ = 0.1488 nm) and 6A (λ = 0.1500 nm) of Photon Factory in KEK (Japan). The two-dimensional SAXS intensity was detected using PILATUS3 2M and 1M. A series of X-ray structure analyses were performed using a homemade GUI software^45^.

## Electronic supplementary material

Supplementary Information
