# Supplementary material for: Filamentous Virus-based Assembly: Their Oriented Structures and Thermal Diffusivity
Source: Sci Rep. 2018 Apr 3;8:5412. doi: 10.1038/s41598-018-23102-1 (PMC5883014; doi:10.1038/s41598-018-23102-1)
Supplement: Supplementary file 1 — Supplementary Information [file 41598_2018_23102_MOESM1_ESM.pdf]

Filamentous Virus-based Assembly: Their Oriented Structures and Thermal Diffusivity

Toshiki Sawada,*[1,2] Yuta Murata,[1] Hironori Marubayashi,[1] Shuichi Nojima,[1] Junko Morikawa,[3] Takeshi Serizawa*[1]

[1] Department of Chemical Science and Engineering, School of Materials and Chemical Technology, Tokyo Institute of Technology
[2] Precursory Research for Embryonic Science and Technology, Japan Science and Technology Agency
[3] Department of Materials Science and Engineering, School of Materials and Chemical Technology, Tokyo Institute of Technology
E-mail: tsawada@polymer.titech.ac.jp, serizawa@polymer.titech.ac.jp

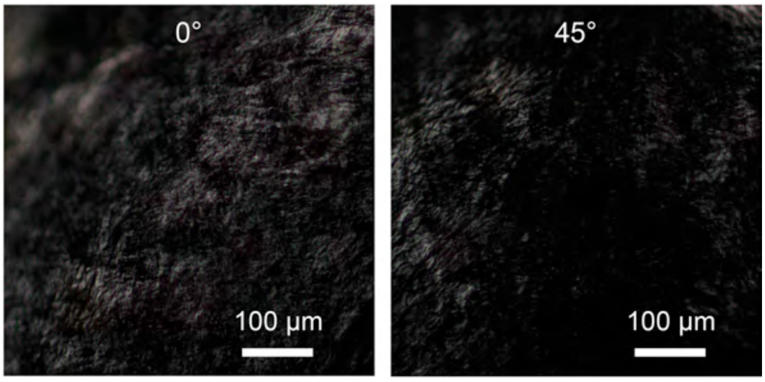

Figure S1| POM images of the non-oriented phage films prepared using simple cast methods.

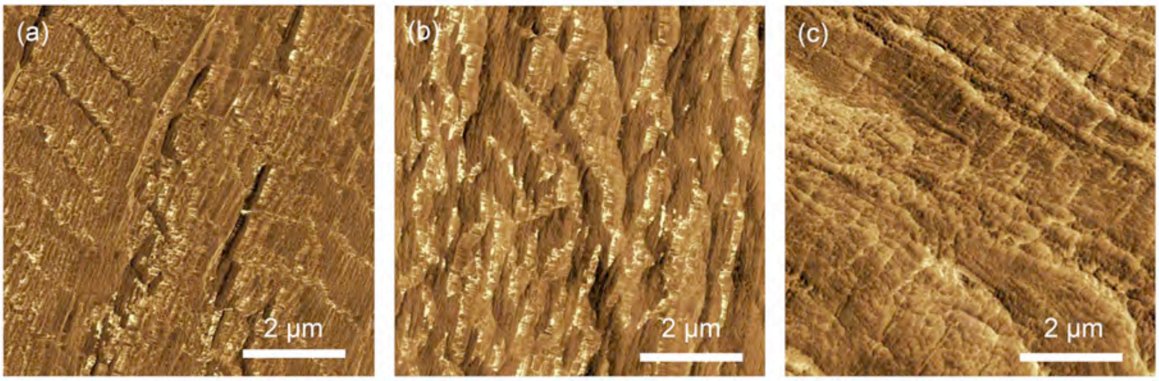

Figure S2| AFM phase images of the (a) outside, (b) midpoint, and (c) center of the phage films.

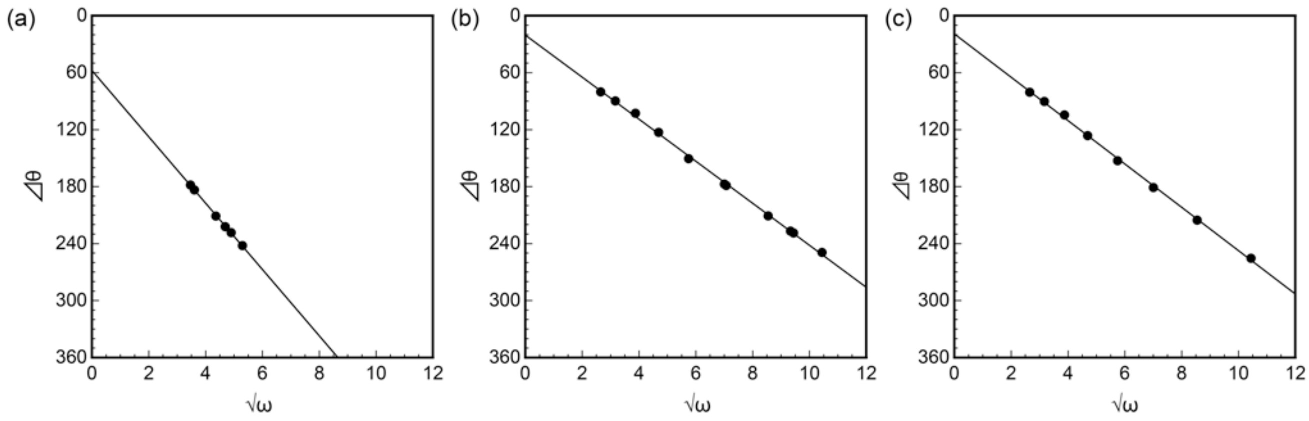

Figure S3| Thermal diffusivity measurements of the (a) outside, (b) midpoint, and (c) center of the phage films. The thicknesses of the films were (a) 181.0, 43.0, and 45.8 μm, respectively.

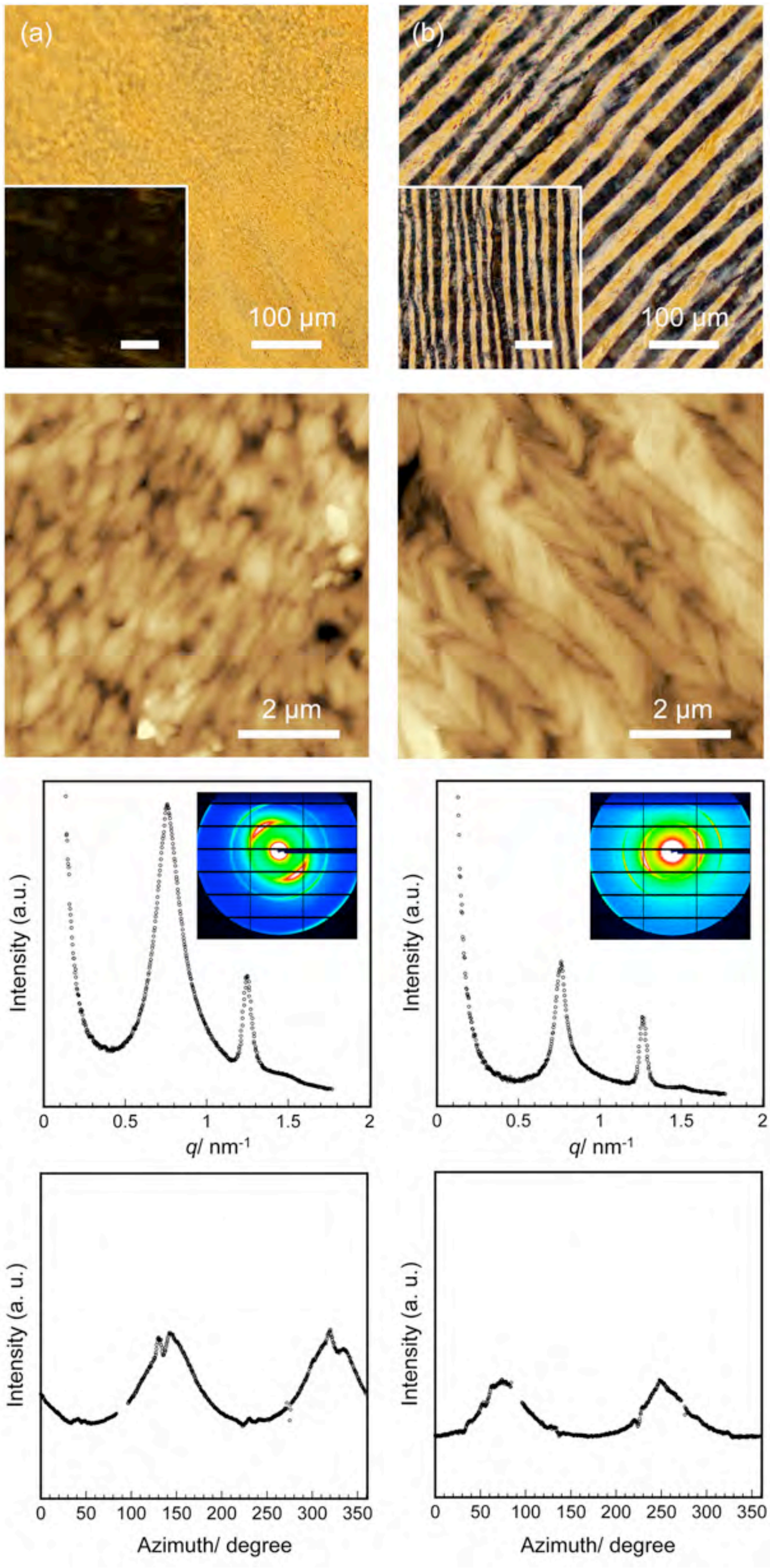

Figure S4| Structural characterization of the previously reported phage films prepared in microtubes. (**a**) Nematic and (**b**) smectic liquid crystalline-oriented POM, AFM, SAXS, and azimuth scan of the primary peaks of the SAXS profiles are shown.
